# Myelodysplastic syndrome progress to acute myeloid leukemia: new insights and updates

**DOI:** 10.3389/fimmu.2026.1769944

**Published:** 2026-02-04

**Authors:** Yucheng Zhang, Lixiang Yan, Chenyang Fan, Bofan Zhao, Meng Chen, Xiaogang Hao, Gengda Zhu, Yanan Jia, Yajing Xu, Zhexin Shi

**Affiliations:** 1 Department of Hematology, First Teaching Hospital of Tianjin University of Traditional Chinese Medicine, Tianjin, China; 2 National Clinical Research Center for Chinese Medicine, Tianjin, China; 3 Pediatrics of Traditional Chinese Medicine, Beijing Jingdu Children’s Hospital, Beijing, China

**Keywords:** acute myeloid leukemia, acute myeloid leukemia with myelodysplasia-related changes, bone marrow microenvironment, myelodysplastic syndrome, myeloid neoplasm

## Abstract

The progression of myelodysplastic syndromes (MDS) to secondary acute myeloid leukemia (sAML), classified under AML with myelodysplasia-related changes (AML-MRC), is a multi-step process driven by the dynamic interplay between cell-intrinsic genetic events and extrinsic microenvironmental remodeling. In this review, we discuss how these changes foster clonal selection and leukemic transformation. Emerging insights from single-cell technologies are highlighted, revealing the dynamic heterogeneity of MDS stem cells and their niche. Finally, we discussed the clinical implications of these mechanisms, including their impact on risk stratification, therapy failure (particularly after hypomethylating agents), and the development of novel treatment strategies aimed at intercepting progression. Integrating molecular findings with clinical translation is essential for improving outcomes in this high-risk disease continuum.

## Introduction

Myelodysplastic syndromes (MDS) represent a group of heterogeneous clonal disorders characterized by ineffective hematopoiesis and distinct morphological abnormalities in the bone marrow (1). As part of a continuum of myeloid neoplasms (MNs), MDS shares considerable homology with acute myeloid leukemia (AML) (2). Throughout the clinical course of MDS, factors such as treatment failure, prolonged disease duration, and other unpredictable elements frequently contribute to disease progression, typically manifested by worsening hematopoietic failure and an increasing percentage of blasts. The pathogenesis of MDS is multifactorial, involving a complex interplay of genetic, epigenetic, and microenvironmental factors (3). Disease transformation is defined by an increase in blast count exceeding 20% in the bone marrow (BM), at which point the condition is classified as secondary acute myeloid leukemia (sAML), specifically categorized under the current World Health Organization (WHO) and International Consensus Classification (ICC) systems as AML with myelodysplasia-related changes (AML-MRC). According to the latest ICC guidelines (4), AML-MRC is further stratified into three distinct subtypes: AML with mutated TP53, AML with myelodysplasia-related gene mutations, and AML with myelodysplasia-related cytogenetic abnormalities (5).

In 2020, Andrew J. Menssen and Matthew J. Walter provided a comprehensive overview of the genetic basis underlying progression from MDS to secondary leukemia (6), highlighting the necessity for future research utilizing RNA-Seq and other advanced technologies. It is now well-established that the transformation from MDS to sAML is driven by a complex interplay involving the BM immune microenvironment, the stepwise acquisition of additional driver gene mutations, and epigenetic alterations in hematopoietic stem and progenitor cells (HSPCs) (7). These epigenetic mechanisms encompass DNA methylation, RNA splicing, histone modification, and chromatin organization (8, 9), and share recurrent mutations (10). While MDS and sAML share mutations in at least six key molecular pathways (6), AML-MRC frequently acquires additional mutations in transcription factors and signal transduction genes (10, 11), reflecting late-stage biological events in MDS progression. Current research still lacks a complete understanding of late-stage MDS changes, some studies have yielded conflicting conclusions, and the full biological picture of MDS-to-sAML progression remains unclear. What is evident, however, is that this process is consistently shaped by the bidirectional interaction between stem cell mutations and microenvironmental remodeling.

This review provides an updated synthesis, structuring the discussion around two core, interconnected axes of progression: (1) intrinsic changes within the MDS stem and progenitor cell compartment, and (2) extrinsic remodeling of the bone marrow microenvironment. We critically evaluate areas of conflicting evidence, discuss the clinical translation of molecular insights, and conclude with a framework linking pathogenic mechanisms to risk stratification and therapeutic opportunities.

## Changes of MDS stem cells

The progression of MDS is fundamentally a process of clonal evolution. This section examines the intrinsic cellular and genetic alterations within HSPCs that provide the substrate for malignant transformation. We focus on the modes of clonal expansion, the controversial roles of telomere biology, and the functional impact of key recurrent mutations, integrating single-cell data that reveal the heterogeneity of this process.

### Expansion of clone-driven evolutionary model

Previous studies have indicated that clonal evolution, driven by the acquisition of additional somatic mutations, can promote clonal progression in MDS, thereby revealing dynamic patterns of clonal architecture and evolution (2). Single-cell sequencing studies have been pivotal in deciphering this complexity. At the stem cell level, it has been demonstrated that abnormal MNs cells present within hematopoietic stem cell (HSCs) compartments—commonly defined by immunophenotypic markers—constitute a cellular source of disease progression (12). In nearly all cases, MDS progression is associated with clonal evolution, typically characterized by the expansion or emergence of subclones harboring distinct sets of mutations.

Early work by Chen et al. using longitudinal paired samples revealed that the subclonal diversity within stem cells may be greater than that in more differentiated progenitor cells in both MDS and AML-MRC. The MDS stem cell clone giving rise to AML-MRC is not always the diagnostic dominant clone, indicating non-linear evolution at the stem cell level, which is predominantly associated with malignant transformation (12). This heterogeneity is further exemplified by the identification of distinct immunophenotypic architectures: one driven by common myeloid progenitors (CMPs) and another by granulocyte-monocyte progenitors (GMPs), primarily driven by genetic alterations in *TP53*, *RUNX1*, *DNMT3A*, *BCOR*, and *STAG2*, respectively (13). Wang et al. demonstrated that CMPs constituted the most significant HSPCs subpopulation in MDS patients with excess blasts or MDS-EB/sAML compared to healthy individuals, and highlighted the critical roles of *PLCB1* and *MAML3* in promoting cell proliferation, survival, and AML transformation within CMPs of MDS patients (14), although other studies indicate that pre-leukemic HSCs must differentiate to the GMP stage to acquire a leukemic stem cell phenotype (15). The two immunophenotypes maintain separate transcriptional states and differentiation trajectories throughout progression, highlighting the considerable heterogeneity inherent in the clonal evolution of MDS.

Complementary single-cell DNA sequencing data delineated two broad evolutionary patterns: a “static” group, in which potential epigenetic alterations—such as initial mutations in DNA methylation genes (*DNMT3A, TET2, IDH1/2*)—drove progression to AML-MRC through mutational burden accumulation; and a “dynamic” group, characterized by acquisition of new chromosomal abnormalities, *TP53* or signaling gene mutations, accompanied by substantial restructuring of the clonal architecture (16). Furthermore, The evolutionary pattern of chromosome karyotypes is also highly complex, including branching, linear, parallel, and macro-evolutionary trajectories (17).

Chromatin accessibility includes epigenetic landscapes including the integration activity of enhancers, promoters, and transcription factors that control hematopoietic and cancer development, an approach increasingly applied to study alterations in hematopoietic stem and progenitor cells in MDS (18, 19). This adds another layer, showing that Lin^-^CD34^+^CD38^-^ MDS cells progressively lose stem-like chromatin states and undergo premature myeloid transcriptional activation during advancement, confirms that the chromatin landscape in MDS stem cells governs their autonomous cellular behavior and drives disease progression (20).

The above studies present a spectrum of evolutionary models. Discrepancies may arise from technical differences, patient cohort heterogeneity, and the challenge of capturing a dynamic process from static. The finding of progenitor-derived architecture (13) contrasts with models suggesting a requirement for differentiation to the GMP stage for full leukemic transformation (15). These may not be mutually exclusive but represent different paths within the MDS’s biological heterogeneity. A key limitation is the small cohort size typical of these complex single-cell studies, which may not capture the full diversity of evolutionary routes. Larger, longitudinally sampled cohorts using multi-omics single-cell approaches are needed to resolve the prevalence and context of each trajectory and to determine if specific genetic subtypes favor particular evolutionary modes.

### Telomere biology in clonal selection

Telomere attrition is a recognized hallmark of aging and genomic instability, a significant contributing factor in the pathogenesis of MDS and AML. In MDS, critically short telomeres correlate with higher-risk disease and inferior survival (21). In general situation, a longer shortest telomere length (TL) was associated with improved progression-free survival (PFS) and overall survival (21). Stem cell attrition in the context of short telomeres, compounded by aging, drives clonal evolution. The germline mutations in telomerase and related genes are sufficient to initiate a clonal hematopoiesis -like phenotype (22).

Notably, The family derived from BM accounts for approximately 75% of disorders associated with shortened telomeres (23), which themselves naturally shorten with aging. Short telomere-mediated MDS/AML represents a rare phenotype often presenting as aplastic anemia; regardless of marrow cellularity, patients with this diagnosis typically exhibit cytopenias and frequently follow an indolent disease course characterized by slow-progressing MDS (22). As telomere shortening mutations are commonly inherited in an autosomal dominant pattern, they are readily linked to germline susceptibility syndromes. In the model proposed by Kristen E. et al., stem cell attrition in the context of short telomeres, compounded by aging, drives clonal evolution. Their work demonstrates that germline mutations in telomerase and related genes are sufficient to initiate a clonal hematopoiesis (CHIP)-like phenotype (22).

Conversely, paradoxically, mutations in spliciactors (SRSF2, SF3B1, U2AF1) (24) or the POT1 mutation-related long telomere syndromes demonstrate a notably high prevalence of clonal hematopoiesis (CH) (25), conferring a clonal advantage by circumventing replicative senescence, helping explain the age-related incidence of MDS.

The prognostic significance of telemore also extends to allogeneic hematopoietic stem cell transplantation (allo-HSCT) for MDS. A longer donor leukocyte TL is associated with superior 2-year survival and reduced non-relapse mortality. Furthermore, greater leukocyte TL shortening in recipients at three months post-HCT is correlated with a lower risk of disease relapse (26). Given this evidence, targeting telomerase to modulate telomere maintenance is emerging as a promising therapeutic strategy. The novel telomerase inhibitor imetelstat, effectively impairs malignant HSCs and leukemic stem cells (LSCs) by disrupting telomere maintenance, while largely sparing normal cells. This agent has demonstrated significant clinical activity in improving erythropoiesis in patients (27).

This presents a clear dichotomy: both excessive shortening and aberrant lengthening/maintenance of telomeres can promote clonal outgrowth. This underscores that dysregulation of telomere homeostasis—rather than simply its shortening—is oncogenic. The germline susceptibility vs. somatic mutation in splicing factors, and the accompanying genetic landscape likely determine this effect. These findings challenge a linear model where telomere shortening is the sole driver of HSC aging and clonal selection, introducing a more nuanced view where specific mutations actively rewire telomere biology to gain a fitness advantage.

### Mutations in genes and epigenetic modifiers

The genetic landscape of MDS is recognized as a principal driver of disease progression, these alterations profoundly affect HSCs function and lineage (28). Although mutations strongly associated with AML *de novo*—such as in NPM1, CEBPA, or MLL rearrangements—are considered rare in early-stage MDS, however, specific genomic changes present at diagnosis can still alter disease biology and accelerate progression. For example, mutations in spliceosome genes (SRSF2, SF3B1, U2AF1), EZH2, BCOR, and STAG2 strongly suggests an MDS origin for some AMLs (6). In some cases, the transformation to AML-MRC requires additional oncogenic hits (29). As we can see, a 2024 proposal for the molecular classification of MDS introduced a new paradigm, using genetic subtypes as the primary framework for classification, with blast count defining the disease stage within each molecular subgroup (30).

Here, we focus on selected mutations with established roles, summarizing their frequencies and proposed mechanisms.

### STAG2

The cohesin STAG2 is important in myeloid differentiation and is necessary for the exit from the most primitive stem and progenitor cell compartment. Along with EZH2 and ASXL1, it is highly correlated with trisomy 8 (31) and are associated with shorter survival, ranking them among the most frequent cohesin alterations in this disease (32). Mutations in STAG2 occur in nearly 10% of MDS patients, the detection of a STAG2 mutation in an AML patient often implies a preceding MDS phase, highlighting its utility as a diagnostic marker for disease ontogeny (10).

The mechanistic link between STAG2 loss and leukemic progression is actively being delineated. Recent functional CRISPR screens have demonstrated that *STAG2* loss leads to accumulation of HSPCs alongside a reduction and impaired differentiation of mature cells (33). Furthermore, emerging evidence suggests that STAG2 mutational status may influence response to HMAs, the cornerstone of MDS therapy, though this effect requires further validation (34). However, these divergent findings may be influenced by the biological heterogeneity of cohesin complex components and their specific genetic contexts, where different co-mutation patterns can concurrently confer either favorable or adverse prognostic effects.

### Transcription factors (*RUNX1, GATA2*)

Mutations in the transcription factor *RUNX1*, essential for normal hematopoiesis, are found in approximately 10% of MDS cases, associated with loss of chromosome 7 or 7q, a high risk of transformation to AML-MRC, and inferior overall survival (35). *RUNX1* mutation often acts as a secondary genetic hit during progression, often serving as a secondary genetic hit during progression from MDS to AML (36). In low-risk MDS, *RUNX1* mutation impairs DNA repair and induces senescence in CD34^+^ cells, producing a transcriptional state akin to high-risk disease (35). Its cooperative effects are significant: co-occurrence with *SRSF2* mutation induces multilineage hematopoietic defects (37), and synergy with miR-146a deletion drives dysregulated innate immune signaling that can precipitate MDS and eventual AML (38). These studies collectively confirm that *RUNX1*, even when present early in MDS, can significantly alter disease biology and accelerate its progression.

*GATA2* is a master regulator of hematopoietic development, is a major predisposing factor for childhood MDS. Germline *GATA2* mutations lead to cytopenias, with over 80% of carriers developing MDS by early adulthood, a condition frequently marked by excess blasts and progression to sAML (39, 40). The disease biology in this context is shaped by specific co-mutations. *ASXL1* and *STAG2* are frequently co-mutated in *GATA2*-deficient MDS (41). While mutations in *SETBP1* or *ASXL1* alone may not severely impair differentiation, they synergize with *GATA2* deficiency to disrupt myeloid development *in vitro*, with the triple combination of *GATA2* loss, *SETBP1*, and *ASXL1* mutations causing the most severe hematopoietic disruption (42).

### TET2

*TET2* is considered central to the pathogenesis of MDS/AML (43). As a key epigenetic regulator and an enzyme involved in active DNA demethylation, *TET2* mutations are common in both MDS and AML. *TET2* inactivation in MDS leads to increased IL-6 expression (44), promotes NLRP3 inflammasome activation and IL-1β production (45), and fosters an inflammatory BMME that drives sustained immunosuppression (46). Furthermore, stress-inducing factors such as infection can promote the selection of *TET2*-deficient HSCs. Through the Trif-Plk-Elf1 axis, *TET2* loss reshapes the transcriptional and epigenetic landscape of HSCs, potentially enabling MDS development even without increasing the genomic mutation burden (47). In *TET2*-deficient HSPCs, overactivation of IL-6 rendering these cells resistant to cytokine-induced apoptosis (48). Recent work also shows that *TET2* deficiency impairs natural killer (NK) cell function by altering methylation patterns and reducing the expression of cytotoxic effector molecules, facilitating immune escape and disease persistence (49).

### Splicing factors (*SRSF2, U2AF1*)

*SRSF2* is involved in both constitutive and alternative splicing, facilitating the assembly of spliceosomal complexes (50). Mutations in *SRSF2* are common recurrent alterations in MDS. In MDS/AML harboring *SRSF2* mutations, aberrant splicing caspase 8 occurs in HSPCs, disrupting the regulation of pyroptosis, apoptosis, and necroptosis (51), leading to significant increased mitophagy compared to other subtypes.

In 2015, Kim et al. demonstrated that *SRSF2* mutations directly impair hematopoietic differentiation *in vivo*, an effect not solely attributable to loss of its normal function, but drives recurrent mis-splicing of key hematopoietic regulators such as *EZH2*, resulting in compromised differentiation (52). Jane et al. also confirmed that *SRSF2* is a somatic mutation (SM) associated with the increased risk of AML progression directly or indirectly from LR-MDS (53).

Mutations in splicing factors *SRSF2* and *U2AF1* can also act synergistically. These mutations converge to activate G-protein and ERK/MAPK signaling, which drives MDS and renders the mutant cells sensitive to MEK inhibition (54). Similarly, *ASXL1* and *SRSF2* are frequently co-mutated in MNs. This co-mutation accelerates disease development, increases severity, and hastens transformation to AML-MRC (55), conferring distinct genetic features and poorer survival compared to patients with single or no mutations (56). *SRSF2* mutations also induce aberrant splicing of mitochondrial genes, leading to mitophagy and metabolic dependency (57). Liu et al. further identified aberrant *PINK1* splicing as a specific vulnerability in *SRSF2*-mutant AML and MDS, providing a rationale for developing targeted spliceosome inhibitors. Notably, in the context of HMA failure, *U2AF1* mutations have been associated with better outcomes, highlighting context-dependent roles (58).

### Tp53

*TP53* is a tumor suppressor gene located on chromosome 17p that encodes the transcription factor p53. MDS and AML cases with *TP53* mutations are typically associated with complex karyotypes (CK) and poor prognosis (4). *TP53* Mutations, especially biallelic inactivation, define a distinct entity with complex karyotypes, profound immune dysregulation, and a very high risk of transformation, recent work consolidates that *TP53*-mutant MDS and AML represent a biological continuum (59–61). Within the MDS course, evolution from monoallelic to multi-hit status often coincides with AML progression, and treatment itself also may provide a selective pressure favoring *TP53*-mutant clones. For instance, lenalidomide has been associated with the emergence or expansion of multi-hit *TP53* clones in a subset of patients (62, 63).

A key 2025 study analyzing 6,204 MDS patients, demonstrating that MDS with p53 dysfunction displays profound immune dysregulation involving myeloid inflammation and impaired antigen presentation. This study observed an increased frequency of immunosuppressive Tregs within the CD4^+^ compartment and a higher proportion of exhausted PD-1^+^TIM3^+^ cells alongside Granzyme B/K^+^ double-positive effector cells among CD8^+^ T cells. These cases showed downregulation of MHC class II genes and broader pathways of innate and adaptive immunity, while pathways related to oxidative stress, transcription, and MYC targets were upregulated (63). These findings align with and extend previous work, collectively explaining the biological basis for the uniformly poor prognosis associated with *TP53* mutations in MDS/AML.

The prognostic value of these mutations is scored in systems like IPSS-M. However, their predictive power for transformation remains imperfect. The mutation frequency data presented in **Table 1**, sourced from public portals like cBioPortal, offer population-level insights but are subject to selection biases inherent in the original datasets. These frequencies may not reflect the true prevalence in an unselected MDS population, has its own potential limitations. Furthermore, the functional consequence of a mutation is modulated by its clonal architecture and co-mutation context. This complexity necessitates integrative models beyond single-gene for accurate risk prediction.

**Table 1:** Recent studies on MDS somatic mutations promoting the progress of MDS.

Mutations	Frequencies	Function	Possible biologic mechanism
STAG2	6.61%	Cohesin	Accumulation of HSPCs; impairment of hematopoietic differentiation; Demethylation resistance
TET2	22.97%	DNA demethylase	Abnormal methylation; upregulation of IL-6, NLRP3, and IL-1β expression drives immune suppression
GATA2	2.52%	Transcription factor	Induce hematopoietic destruction, act as a somatic driven mutation of early MDS in child
RUNX1	11.60%	Transcription factor	Correlation with -7/7q leads to MDS; Induce multiple lineage hematopoietic defects, lead to DNA damage repair disorder and cell aging
SRSF2	13.13%	RNA splicing & selection	Cause mRNA decay; Damage hematopoietic differentiation; Coordination with ASXL1/U2AF1-SRSF2 co mutation
TP53	10.52%	Tumor suppressor	Related to the CK; Keep accumulate in the process of to drive MDS evolution

The mutation frequency data was obtained from cBioPortal (accessed Dec 6, 2025). Mutation frequency represents the number of patients with at least one mutation in the specified gene divided by the total number of patients analyzed in each dataset, as reported in cBioPortal. A total of 7583 samples were obtained. MDS datasets include: Myelodysplasia (Utokyo, Nature 2011); Myelodysplastic (MSK, 2020); Myelodysplastic Syndromes (MDS IWG, IPSS-M, NEJM Evidence 2022).

## Pathological remodeling of the bone marrow microenvironment

The progression of MDS is not solely determined by intrinsic genetic changes within hematopoietic cells. A permissive and actively supportive bone marrow microenvironment (BMME) is a critical basement. Various cellular components within the BMME can directly participate in MDS progression (64). The emergence of leukemic clones arises from the selective advantage conferred to transformed HSPCs within the context of BMME alterations and inflammatory stimuli. This section examines how non-hematopoietic stromal components and immune cells are pathologically altered in MDS, creating a niche that fosters clonal evolution, suppresses anti-tumor immunity, and ultimately facilitates leukemic transformation.

### Bone marrow stromal cells

BMSCs are the non-hematopoietic cellular component of the marrow, constitute approximately 20% of the cellular volume in normal BM and form the direct physical and functional niche where HSPCs reside and develop (65). In MDS, this supportive niche becomes pathologically remodeled.

A study utilizing single-cell RNA sequencing revealed an inflammatory reprogramming within the stromal stem cell niche of patients with LR-MDS (66). This remodeling is driven by infiltrating pro-inflammatory CD8+ T cells that exhibit high levels of activation markers like TNFRSF9, CD69, HLA-DRA and secrete cytokines such as TNF-α, IFN-γ, CCL3, and CCL4. Critically, this inflammatory shift occurs independently of specific genetic drivers in the hematopoietic clone. It promotes clonal evolution by conferring a relative survival advantage to mutated HSPCs, making them resistant to the otherwise inhibitory inflammatory signals, thereby linking stromal inflammation directly to leukemic progression risk (66). This transition is likely mediated by soluble factors including IFN-γ, TNF-α, and IL-6 (67). Furthermore, NF-κB activation in BMSCs leads to the sustained upregulation of inflammatory factors and hematopoietic inhibitors such as IL-6, IL-8, and CCL-3, associated with a reduced capacity of BMSCs to support healthy HSPCs, illustrating a direct functional impairment of the niche in LR-MDS (68).

### Mesenchymal stem cells

MSCs are essential regulators of the HSC niche. In MDS, MSCs undergo functional and transcriptomic alterations, changing from supporters of normal hematopoiesis to enablers of dysplastic and leukemic clones.

The dysfunction of MSC in MDS manifests as senescence, inflammatory, reduced differentiative potential, and altered metabolism. Key drivers include cytokine signaling like IL-1β/IL-18 inducing senescence (69) and intrinsic metabolic defects. Functionally, MDS-derived MSCs lose their preferential support for healthy HSPCs while enhancing the engraftment and survival of MDS cells (69–71). Some of these defects may be intrinsically driven by MDS MSCs themselves. For example, MDS MSCs produce exosomes enriched in CPT1A, which disrupt their own metabolic homeostasis, thereby impairing their ability to support normal HSCs and promoting ineffective hematopoiesis (72). As disease advances, MSCs in higher-risk subtypes secrete more inflammatory factors, promote myeloid-skewed differentiation of HSPCs, and exhibit resistance to HMAs (73). Although recent studies indicate that alterations in MSCs from MDS patients lack evidence of clonal mutations (74), these changes still pointing to a reactive, environmentally-driven dysfunction (75).

The pathogenic role of MSCs is underscored by their potential as biomarkers and therapeutic targets. The content and gene expression profile of MSCs may predict AML transformation (76). Genes like HOXB3 and HOXB7, progressively upregulated in MSCs during progression, represent novel therapeutic targets; their inhibition can partially restore normal MSC function (77). Furthermore, drugs like eltrombopag has recently been shown to ameliorate the inflammatory status of the BMME in LR-MDS and restore the self-renewal capacity and adipogenic-osteogenic differentiation balance of MSCs, suggesting microenvironment-directed therapy is feasible (78).

However, studies on BMSCs/MSC senescence, inflammatory secretion, and metabolic defects often focus on different subsets or disease stages, making a unified model challenging. Most evidence is derived from *in vitro* co-culture or xenograft models, which may not fully recapitulate the human BMME complexity. Furthermore, the causal relationship between BMSCs/MSC alterations and *in vivo* specific progression requires more rigorous validation.

### Myeloid derived suppressor cells and immune cells

The establishment of a severe immunosuppressive BMME is a hallmark of MDS progression, primarily driven by the expansion and activation of MDSCs and their interplay with dysregulated immune cells populations.

MDSCs constitute a distinct population of myeloid-derived cells with potent immunosuppressive functions, setting them apart from conventional macrophages (79). In the inflammatory MDS-BMME, factors such as IL-8 overexpression drive the recruitment and expansion of MDSCs (80). These cells exhibit a potent immunosuppressive phenotype, characterized by elevated secretion of IL-10 and TGF-β1 (81). A critical self-reinforcing survival mechanism involves an autocrine IL−6-driven *STAT3–DNMT* epigenetic axis that silences TNF−α expression, facilitating their persistent accumulation (82) and fostering a niche resistant to the programmed death-1 (PD-1) targeted therapy (83). The immunosuppressive potential of these cells is further reflected in the IL-10/IL-12 and TGF-β/TNF-α ratios, correlates with higher-risk disease and poorer prognosis (84), underscoring the prognostic relevance of MDSCs in disease aggravation.

MDSCs do not act in isolation. Recent investigations have highlighted the unique role of Tregs in the immune dysregulation of MDS. The MDSCs abundance is positively correlated with an increase in Tregs, part of a broader immunosuppressive shift by an increase in Th2 and Treg populations alongside a decrease in Th1 and Th17 cells (85). Recent studies indicate that in AML patients with elevated IFN-γ, immune exhaustion and escape may result from leukemic cell–MSC interactions that drive Treg expansion (86). In contrast, in low-risk MDS, although a pro-inflammatory cytokine milieu promotes apoptosis, Treg-mediated immunosuppression is not yet predominant. Nevertheless, Zhang et al. identified differentially expressed cytokines—including TNF-β, IL-9, and β-NGF—in low-risk compared to high-risk MDS, suggesting early involvement of immune dysregulation that may drive pro-inflammatory and pro-apoptotic signaling (87). This axis collectively suppresses cytotoxic T-cell and natural killer (NK) cell function, enabling immune escape of the malignant clone (88). Dendritic cells (DCs) and monocytes are also functionally impaired in MDS, displaying a reduced capacity to prime effective anti-tumor T-cell responses, further crippling adaptive immunity (89). The immunosuppressive activity of MDSCs extends beyond the BM and is also operative in the peripheral blood. Anne et al. (90) reported that peripheral blood MDSCs exhibit potent immunosuppressive activity from day +30 following allo-HSCT. These cells displayed activated NLRP3 inflammasome signaling, which contributed to impaired donor immune effector functions and correlated with early relapse post-transplantation. Collectively, growing evidence suggests that MDSCs may be implicated throughout the multistep pathogenesis of MDS—from the initial emergence of malignant clones through clonal evolution, immune evasion, and ultimately, treatment resistance (91).

Targeting the MDSCs-T cell network is a major translational therapeutic frontier. Strategies include direct modulation of MDSCs, such as valproic acid combined with anti-PD-L1 (92) and the use of bispecific antibodies like CD123-directed to engage T cells against both blasts and MDSCs (93). HMAs like decitabine may also reduce MDSCs accumulation (82). Thus, targeting MDSCs represents a promising therapeutic strategy for MDS/AML, although following these strategies has proven difficult. Some early-phase clinical trials targeting immune checkpoints have failed to show significant modulation of the MDSC compartment or substantial clinical benefit (94), underscoring the complexity and redundancy of immune evasion mechanisms in MDS.

**Figure 1:**
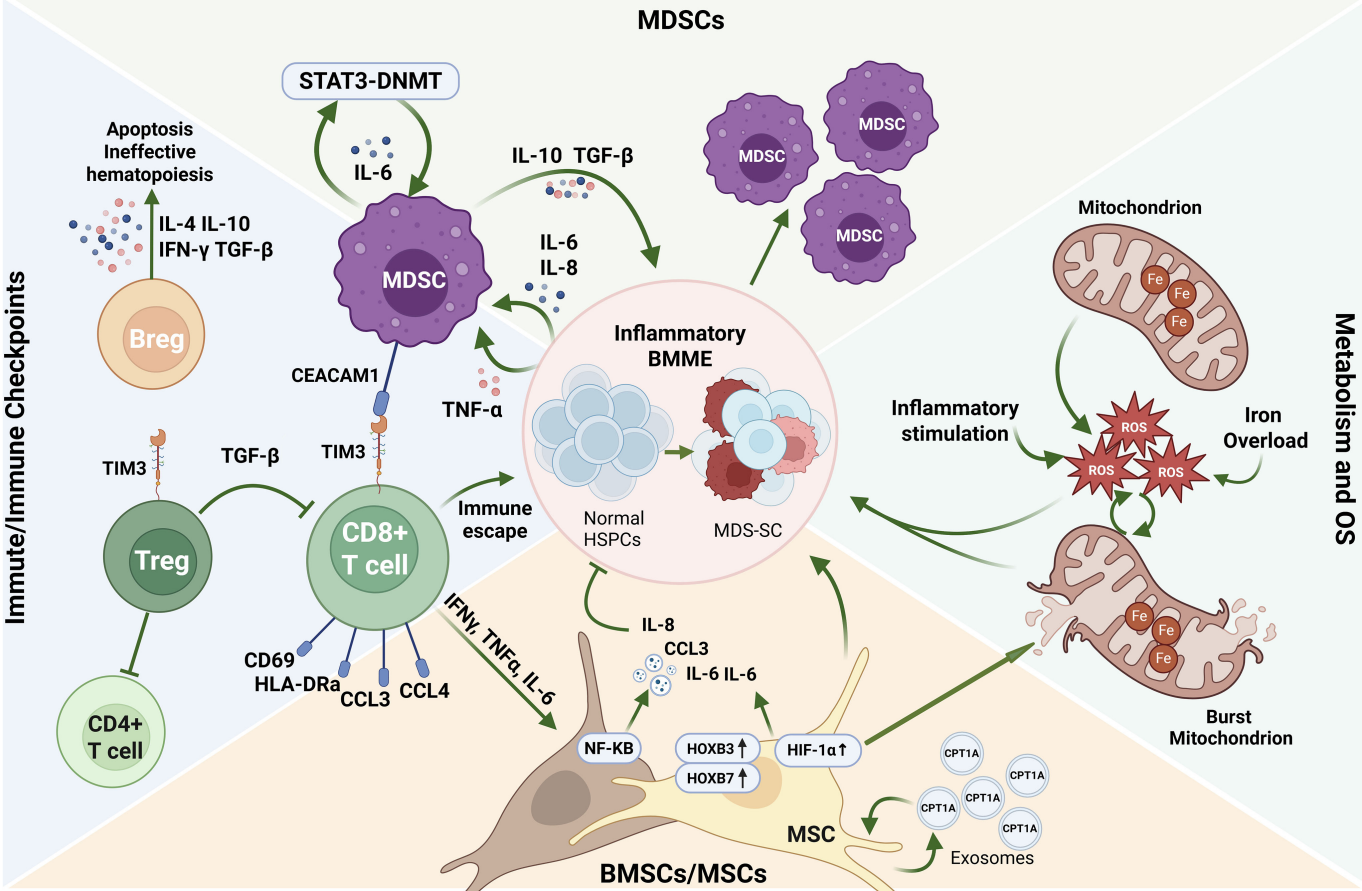
Parallel clonal evolution model from MDS to AML-MRC. This figure illustrates the heterogeneous, multi-step transformation of MDS to AML with AML-MRC, encompassing both linear and parallel evolutionary paths at the stem and progenitor cell level. Within a context of genetic susceptibility, aging, and abnormal telomere biology, HSPCs acquire early driver mutations, leading to CH and pre-MDS-SCs. These subsequently evolve into distinct MDS-SC subsets with different immunophenotypes. Early key driver mutations, such as in TET2 and DNMT3A, establish the initial clone, while later genetic events including secondary TP53 mutations and lesions in RUNX1, PLCB1, or MAML3 drive progression toward a more aggressive phenotype. This evolution culminates in the emergence of AML-MRC stem cells and the expansion of blasts. Available evidence indicates that evolutionary states involving CMPs and GMPs lineages may coexist in the same patient, underscoring the diversity of clonal evolutionary model in this disease. Created with BioRender.com (https://app.biorender.com). HSPCs, Hematopoietic Stem and Progenitor Cells; CH, Clonal Hematopoiesis; MDS, Myelodysplastic syndrome; AML-MRC, Acute Myeloid Leukemia with Myelodysplasia-Related Changes; SC, Stem Cells; CMPs, Common Myeloid Progenitors; GMPs, Granulocyte-Monocyte Progenitors.

Notably, TGF-β1—frequently secreted by MDSCs—has been demonstrated to induce BM inflammatory states and can even provoke bone marrow failure independently of genetic drivers (95). Furthermore, T-cell subsets undergo profound alterations in MDS and are modulated through a variety of newly identified pathways. Beyond T cells and MDSCs, B-cell development is also abnormal in MDS. Processes such as antigen receptor-mediated signaling and B-cell activation are suppressed (96), while type I interferon signaling is upregulated, reflecting broader immune dysregulation. Immunosuppressive regulatory B cells (Bregs) with a CD25^+^CD86^+^ phenotype are increased in MDS and may facilitate immune escape of abnormal clones. These Bregs enhance release of cytokines such as IL-10, TGF-β1, IL-4, and IFN-γ, accelerating ineffective hematopoiesis and excessive apoptosis of hematopoietic cells (97). The recently discovered changes in the BMME are depicted in **Figure 1**. In the following section, we will review recent studies on systemic inflammatory and immune abnormalities that are not precisely located in BM.

### Inflammation and immune dysregulation

Beyond localized BM changes, MDS is increasingly recognized as a disorder of systemic immune dysregulation. Chronic inflammation acts as a sustainer of disease, shaping a pathogenic continuum from early clonal expansion to late-stage immune escape. This section examines the role of persistent inflammation, the dynamic evolution of the immune landscape, and the therapeutic implications of key immune checkpoints.

### Chronic inflammation as a driver of clonal selection

**Figure 2:**
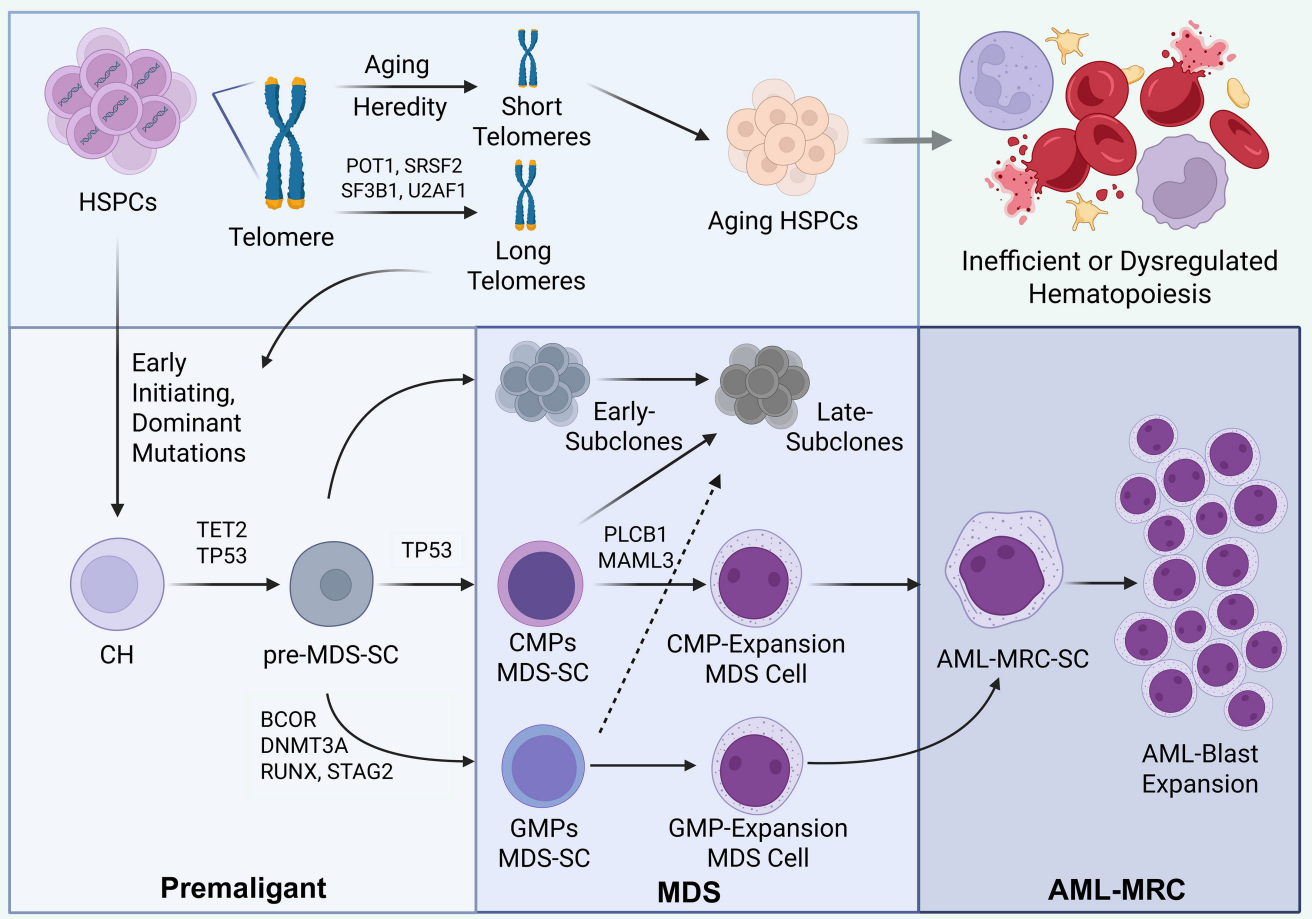
BMME changes in the progress from MDS to AML-MRC discovered by the latest research. This figure outlines the core pathological network within the BMME that drives the transformation of MDS to AML, centered on three interconnected processes: immunosuppression, inflammatory activation, and metabolic dysregulation. MDSCs expand in response to inflammatory signals and sustain their survival via a STAT3−DNMT axis. In concert with TIM3^+^ Tregs and other suppressive immune cells, they establish profound immunosuppression through factors such as IL−10 and TGF−β, enabling immune escape of malignant clones. Simultaneously, BMSCs/MSCs become activated in MDS and, via the NF−κB pathway, release large quantities of pro−inflammatory cytokines including IL−6 and IL−8. This exacerbates inflammation and impairs their normal hematopoietic support function. Furthermore, inflammation coupled with iron overload promotes mitochondrial dysfunction, leading to excessive ROS production and oxidative stress. This not only directly drives genomic instability and clonal evolution but also forms a self−reinforcing feedback loop with the immune and inflammatory changes described above. Together, these dynamic and intertwined pathological processes create a permissive niche that supports the survival, expansion, and eventual leukemic transformation of malignant clones. Created with BioRender.com (https://app.biorender.com). BMME, Bone Marrow microenvironment; HSPCs, Hematopoietic Stem and Progenitor Cells; MDS, Myelodysplastic syndrome; SC, Stem Cells; BMSCs, Bone marrow stromal cells; MSCs, Mesenchymal Stem Cells; MDSCs, Myeloid-Derived Suppressor Cells; ROS, Reactive Oxygen Species.

Persistent inflammation is a fundamental contributor to the progression of MDS, including aging-related hematopoiesis, clonal hematopoiesis, and cytopenias (98), and may serve as a potential trigger for MDS/AML development (99). Supporting this concept, impaired IFN-γ signaling can promote the outgrowth of DNMT3A-deficient clones by reducing their stress-induced apoptosis (100). This inflammatory background, potentially stemming from aging, metabolic disorders (e.g., obesity, diabetes), or concurrent autoimmune conditions, directly damages HSPCs through reactive oxygen species (ROS) and inflicts DNA damage, thereby accelerating genetic instability and clonal evolution (101, 102). A critical regulatory node is Caspase-8, whose downregulation in MDS-HSPCs under inflammatory stress disrupts cell death pathways and contributes to their dysfunction (51). Thus, inflammation and immune dysregulation function as a “third-hit” mechanism that collaborates with genetic and epigenetic lesions to fuel disease progression (103, 104). This process can be summarized as follows: as MDS advances, the immune landscape within the BMME shifts from a Th1-dominant response toward a state of immune exhaustion. Infiltrating autoimmune lymphocytes become functionally impaired, and immune surveillance progressively declines in adaptation to the chronic inflammatory milieu. This immunosuppressive environment ultimately supports the survival and clonal evolution of myeloid neoplasm cells (101, 103–105). Under the selection of BMME, the complexity demonstration of telomere physiology and clonal evolution can be seen in **Figure 2**.

### Dynamic shift of the immune landscape

Immune dysfunction plays a key role in the pathogenesis of MDS. In 2022, Peng et al. framed the immune imbalance in MDS through the “Yin–Yang theory” from the traditional Chinese Medicine theory, conceptualized as a shift from a state of hyperimmunity to immunosuppression (106). In LR-MDS, the environment is often pro-inflammatory and pro-apoptotic, characterized by active but ultimately ineffective immune surveillance. As the disease advances to HR-MDS and AML-MRC, a state of dominant immunosuppression and immune escape emerges. This shift is marked by T-cell exhaustion, expansion of immunosuppressive MDSCs and Tregs, and functional impairment of antigen-presenting cells like dendritic cells (93, 99, 107). This dynamic model explains the differential therapeutic responses observed: LR-MDS often responds to immunosuppressive therapy, while HR-MDS potentially requiring immune checkpoint inhibition for activation (106).

### Immune checkpoints and therapeutic targets

The immune checkpoint receptor T-cell immunoglobulin and mucin domain 3 (TIM3) is a central regulator of immune dysregulation in MDS, implicated in maintaining LSCs and driving T-cell exhaustion (108). On malignant stem cells, TIM3 expression marks a population with distinct biological properties: despite morphological similarity to TIM3^-^ cells, TIM3^+^ MDS stem cells exhibit lower colony-forming capacity, more karyotypic abnormalities, and a block in differentiation coupled with increased proliferation and apoptosis resistance, suggesting they represent a more aggressive, transformation-prone clone (109, 110). Within the immune compartment, TIM3 is also highly expressed on Treg cells in MDS, associated with TGF-β secretion (85), which weakens control over effector T cells and amplifies overall immunosuppression (111).

Mechanistically, TIM3 interacts with ligands such as galectin-9 (Gal-9), and carcinoembryonic antigen-related cell adhesion molecule 1 (CEACAM1). The TIM3/Gal-9 axis on MDS stem cells can directly induce CD4^+^ T-cell exhaustion, promoting immune escape (112). Furthermore, MDSCs expressing both TIM3 and CEACAM1 enhance immunosuppression, driving CD8^+^ T-cell exhaustion and elevating inhibitory cytokines like IL-10 and TGF-β (113). Thus, blocking TIM3 and CEACAM1 with monoclonal antibodies can partially reverse CD8^+^ T-cell exhaustion (113, 114), representing a potential therapeutic strategy currently under clinical investigation, with monoclonal antibodies like sabatolimab showing potential to restore anti-tumor immunity (108).

### Abnormal energy metabolism and growth regulatory pathways

The MDS-BMME is characterized by profound metabolic dysfunction. In this section we will discuss how dysregulated energy metabolism, mitochondrial defects, and resultant oxidative stress (OS) create a permissive niche for clonal expansion and MDS progress, intersecting with both genetic instability and immune evasion.

### Oxidative stress

The BMME maintains HSPCs quiescence partly through hypoxia. In MDS, this balance is disrupted, leading to exacerbated OS. ROS is a group of molecular oxygen derivatives primarily generated as byproducts of mitochondrial metabolism during aerobic growth (115). Elevated levels of ROS, primarily from dysfunctional mitochondrial metabolism, are a hallmark of MDS cells and correlate with higher-risk disease and increased DNA methylation (116, 117). This ROS imbalance is not a passive byproduct but an active driver of pathology. It inflicts direct damage through DNA strand breaks, lipid peroxidation, and protein oxidation (118, 119), contribute to clonal evolution and disease progress (120). Moreover, OS can directly impair the activity of epigenetic regulators like TET2, creating a vicious cycle linking metabolic stress to the epigenetic dysregulation central to MDS pathogenesis (45). Given that the relationship between ROS and MDS has been reviewed by Jing et al. in detail previously (115), the following section focuses on recent advances in this field.

### Mitochondrial dysfunction and metabolic reprogramming

Mitochondrial integrity is essential for the self-renewal of both normal HSPCs and LSCs (121–123). Recent studies indicate that mitochondrial dysfunction, iron overload (IOL), inflammation, or oncogenic signaling can each elevate ROS levels, inducing cellular damage that drives genomic instability and clonal evolution. DNA strand breaks, base modifications, lipid peroxidation, and protein oxidation are all linked to MDS progression and transformation to AML-MRC (118, 119).In metabolic changing, MDS cells exhibit a shift toward glycolysis alongside defective mitochondrial respiration. This reprogramming is an adaptive response that supports the anabolic demands of proliferating clones and contributes to leukemic transformation (124).

A key mechanism involves the dysregulation of mitochondrial dynamics leads to an increase in ROS levels. In MNs including MDS, altered mitochondrial morphology—particularly excessive mitochondrial fission—is associated with clonal evolution and disease progression (125). In MDS HSPCs, dynamin-related protein 1 (DRP1)-dependent mitochondrial fission leads to excessive ROS production, which in turn activates inflammatory signaling and promotes dysplastic hematopoiesis with impaired granulocyte differentiation (126) as the dysregulated dynamics. Conversely, loss of DRP1 function can deplete HSCs regenerative potential while maintaining a quiescent state (127). Separately, IOL induces ROS production in MSCs and HSPCs in HR-MDS via the Wnt/β-catenin pathway, causing oxidative damage that can accelerate genetic abnormalities and disease progression (128, 129). Building on the understanding of energy metabolism dysregulation due to mitochondrial defects in MDS, mitochondrial transfer has emerged as a novel therapeutic concept. Notably, mitochondrial transfer also plays a significant role in the pathogenesis, progression, and treatment resistance of hematological malignancies, including AML and ALL, positioning it as a promising therapeutic strategy (130).

### Key regulatory nodes

Hypoxia-inducible transcription factor-1 (HIF-1) is a key regulator of LSCs maintenance and propagation (131, 132) and serves as a marker of oxidative stress in MDS, contributing to ineffective hematopoiesis and disease progression. Studies report seemingly contradictory associations: Qu et al. observed significantly elevated HIF-1α expression in MSCs from MDS patients, which was associated with increased cell cycle arrest and apoptosis—a phenomenon potentially linked to hypoxic conditions in the BMME (133). In contrast, Stergiou et al. reported that HIF-1α activity correlates with MDS severity, where abnormal mitophagy and autophagic cell death, modulated by HIF-1α, intensify with higher blast counts in the BM (134). This discrepancy may reflect cell-type-specific roles or differential regulation across disease stages, warranting further investigation.

The mechanistic target of rapamycin (mTOR) pathway functions as a central signaling node, integrating inputs from growth factors, nutrients, and oncogenic signals to coordinate cell growth, metabolism, and homeostasis in HSPCs (135). Dysregulation of the mTOR signaling pathway is frequently reported in MDS and AML, closely linked to disease progression and adverse prognosis. Heightened mTOR signaling, often associated with aging (136), drives anabolic growth and metabolic reprogramming. Specifically, mTOR complex 1 (mTORC1) activation promotes a glycolytic shift and glucose uptake, largely through the regulation of HIF-1α and MYC, thereby supporting the bioenergetic and biosynthetic demands of expanding clones (137). Beyond metabolism, mTOR signaling interfaces with the epigenetic machinery, where it can upregulate the expression and activity of DNA methyltransferase 1 (DNMT1) (138). Consequently, patients exhibiting enhanced mTOR pathway activity alongside elevated DNA methylation levels often experience poorer clinical outcomes (139), positioning mTOR not only as a biomarker of aggressive disease but also as a compelling therapeutic target to disrupt the linked metabolic and epigenetic drivers of progression.

Simultaneously, targeting the coordinated action of mTOR and other growth- and metabolism-related genes that govern cellular homeostasis offers a promising avenue for developing novel therapies against malignant transformation and cancer progression.

The metabolic landscape of MDS is a promising frontier for therapy. However, key challenges remain. The contradictory data on HIF-1α highlight our incomplete understanding of how oxygen sensing adapts during progression. Targeting metabolic pathways like mTOR holds promise, but must be carefully balanced to avoid harming residual normal hematopoiesis. Future efforts can may aim to integrate metabolic biomarkers with genetic data to better predict progression and design therapies that simultaneously target the metabolic dependencies of the clone and correct the pathological microenvironment.

## Clinical challenges and therapeutic implications

A critical endpoint of the MDS pathogenic processes is therapeutic resistance, such as hypomethylating agent failure (HMAF). This section examines the clinical challenge of treatment failure, its associated molecular and immune features, and emerging strategies to overcome resistance, thereby directly linking disease biology to clinical management.

### HMA failure

HMAs (azacitidine, decitabine) are the cornerstone therapy for HR-MDS patients ineligible for HSCT. Despite this, the overall response rate to HMAs in MDS remains below 50%. HMAF defines a pivotal clinical deterioration point, characterized by rapid progression to AML with mOS typically under six months (140, 141). Recent ASH2025 data confirm the prognosis post-HMAF: among 700 treated patients, 41% experienced HMAF, with a median time from diagnosis to HMAF of 11.9 months and only 8.7 months mOS after failure, with salvage immunotherapy showing limited benefit, underscoring the unmet need (142). Recent evidence is beginning to clarify the mechanisms underlying this progression following HMAF.

Patients with HMAF exhibit a distinct molecular profile. While TP53 mutations and complex karyotypes continued to predict poor outcomes consistent with general MDS, the presence of certain splicing factor mutations like U2AF1 associated with lower early mortality (143), highlighting the nuanced predictive value of genetics in treatment resistance. Beyond genetics, the immune microenvironment adapts during HMA therapy. Notably, Geng et al. found that PD-1 expression was markedly upregulated during the first four cycles of HMA treatment, and high on-treatment PD-1 expression independently predicts subsequent AML transformation and poor survival (144). This suggests that HMAs, while targeting the clone, may inadvertently foster an exhausted immune phenotype, contributing to therapeutic escape.

### Mechanisms of HMA resistance

Understanding HMA resistance mechanisms is the key to developing improved strategies. A genome-wide CRISPR-Cas9 screen in the MDS-derived cell line MDS-L have implicated that loss of the E3 ligase TOPORS sensitizes leukemic stem cells to HMAs by impairing DDR and leading to accumulation of SUMOylated DNMT1 (145). This pathway represents a potential target for combination strategies aimed at re-sensitizing cells to HMAs.

Venetoclax, the BCL-2 inhibitor, highly effective in AML therapy (146), the integration of venetoclax with HMAs represents a significant advance for HR-MDS. Response to this combination is shaped by prior therapy and genetics: prior HMA failure, TP53 mutations, and RAS pathway alterations predict poorer outcomes, whereas mutations in splicing factors U2AF1 and epigenetic modifiers may predict better responses (143) (147). Interestingly, mutations in RNA splicing factors, DNA methylation genes, and ASXL1 appeared to correlate with a more favorable response, emphasizes the need for molecularly informed treatment sequencing.

### Allo-HSCT

Allo-HSCT remains the only clinical method that can cure MDS. Its success is profoundly influenced by genetics, particularly TP53 status, recent ASH 2025 data confirm the critical prognostic impact of TP53 in allo-HSCT for HR-MDS. While patients with TP53-wild showed excellent post-transplant survival, outcomes were significantly worse for those with monoallelic or biallelic mutations, with only a 20.5% 2-year OS (148), this reinforces the notion of TP53-mutant MDS/AML as a distinct entity requiring novel transplant or post-transplant strategies. Interestingly, another study revealed that HR-MDS patients fared better proceeding to transplant either untreated or with only first-line therapy, compared to those receiving HMAs followed by intensive chemotherapy. Outcomes with pre-transplant venetoclax-based regimens trended more favorably than with HMAs (149). These findings might prompt a reassessment of HMA sequencing prior to transplant. Nevertheless, HMAs retain distinct value in specific contexts. For AML with myelodysplasia-related gene mutations, regimens containing HMAs or venetoclax achieved significantly higher complete remission rates than standard idarubicin/cytarabine induction (150), reaffirming their unique therapeutic role.

The management of HMAF is the central challenge in HR-MDS. The integration of venetoclax is a major step forward, but predictive biomarkers for this combination require refinement. The observation that HMAs may upregulate PD-1 calls for caution and suggests potential synergy with checkpoint inhibitors (144). Future strategies must use upfront combination therapies like HMA plus venetoclax in biologically defined higher-risk subgroups to forestall HMA resistance, and develop mechanism-based therapy for HMAF. Transplant decision must be increasingly guided by molecular risk, acknowledging that for TP53-mutant MDS, transplant alone is often insufficient for a long OS.

## Conclusion

In summary, the progression of MDS into AML-MRC is a dynamic, adaptive process fueled by the synergistic co-evolution of malignant clones and a pathologically remodeled BMME, rather than a simple accumulation of genetic events. The evidence reviewed in this study highlights two closely-linked axes driving this progression: the intrinsic clonal evolution of HSPCs, driven by driver mutations, epigenetic reprogramming, and telomere biology dysregulation; and the extrinsic pathological remodeling of the BMME characterized by dysfunctional MSCs, an expanded immunosuppressive network, and a state of chronic inflammation and metabolic stress. This synergy between mutated HSPCs and their microenvironment creates a self-reinforcing loop that drives disease evolution, immune evasion, and ultimately, AML transformation.

Recent advancements, particularly employing high-resolution techniques like single-cell sequencing, have refined earlier models. These studies reveal significant heterogeneity in cellular origins and evolutionary trajectories, challenging the notion of a universal sequence (13, 15, 24, 25, 151). A emerging concept is the bidirectional effect between genetic and the microenvironment. Mutations in genes like TET2 or TP53 can actively swift a pro-inflammatory, immunosuppressive BMME, which in turn exerts selective pressure favoring the expansion and evolution of mutant clones. This paradigm is central to understanding sustained MDS progression. Furthermore, the clinical significance of molecular markers demonstrates pronounced context-dependency, where the same mutation may portend different outcomes depending on disease stage or treatment setting (152), underscoring the necessity for integrated analysis. We suggest that this heterogeneity necessitates a shift toward large-scale, multi-omics studies that integrate genomic, transcriptomic, and epigenomic data across diverse populations to define robust molecular subtypes and evolutionary trajectories. Given the complex spatial and signaling networks within the human body, particularly in the BMME, unraveling this integrated pathophysiology presents both a significant challenge and an essential direction for future research.

A critical synthesis of this evidence requires distinguishing long-established knowledge from genuinely novel insights that are reshaping the model of MDS progression. Established foundations include the prognostic impact of adverse cytogenetics, the role of recurrent somatic mutations in pathways like splicing (37, 55, 153) and epigenetic regulation (TET2, DNMT3A) (45, 47, 48, 100), and the concept of clonal evolution under therapeutic pressure. The most transformative recent advances, however, stem from single-cell and spatial multi-omics technologies, which have illuminated previously unknown complexities. These include the profound heterogeneity of cellular origins and evolutionary trajectories, challenging the classic linear progression model (13, 14). Perhaps the most significant emerging concept is the active, bidirectional crosstalk between the clone and its niche, where mutations actively reprogram the microenvironment, which in turn exerts a selective force on clonal composition.

Several key areas remain contested or unresolved. The precise prognostic and mechanistic role of specific mutations like STAG2 is highly context-dependent and requires further definition (34, 41). The exact sequence and triggers for the immune microenvironment’s shift from a pro-inflammatory to an immunosuppressive state are not fully described (106). Furthermore, seemingly contradictory data, such as the variable associations of HIF-1α expression with disease stage, highlight our incomplete understanding of how stress response pathways are differentially regulated across cell types and disease phases (137). New data directly challenge prior assumptions by showing that mutations like U2AF1 can be associated with favorable outcomes in specific contexts like post-HMAF, underscoring that molecular markers cannot be interpreted in isolation.

Translating this mechanistic understanding into clinical practice requires a forward-looking framework. Future risk stratification models must beyond genetically-centric systems like IPSS-M to integrate microenvironmental features, such as immune cell composition such as Treg, immune checkpoint expression to enable more precise prognostication. Therapeutically, overcoming HMAF demands the development of strategies that co-target the malignant clone and its protective niche. This includes exploring rational combinations of HMAs with novel immune checkpoint inhibitors (e.g., anti-TIM3) in biomarker-defined contexts, developing niche-modulating therapies aimed at reversing immunosuppression (e.g., MDSC depletion, MSC modulation) or interrupting key metabolic stress pathways, and optimizing treatment sequences—such as re-evaluating the timing of allogeneic transplantation or upfront venetoclax-based regimens—based on molecular context. For very high-risk subgroups like TP53-mutant MDS, where current therapies remain inadequate, the development of entirely novel therapeutic approaches is urgently needed.

Finally, by translating this integrated understanding of the “clone-microenvironment” network into refined dynamic risk monitoring and innovative combination therapies, we will aspire to alter the natural history of MDS and improve long-term outcomes for patients.

## Glossary

MDS: Myelodysplastic syndrome
AML: Acute Myeloid Leukemia
sAML: secondary Acute Myeloid Leukemia
AML-MRC: Acute Myeloid Leukemia with Myelodysplasia-Related Changes
MNs: Myeloid Neoplasms
HSCs: Hematopoietic Stem Cells
HSPCs: Hematopoietic Stem and Progenitor Cells
TL: Telomere Length
allo-HSCT: allogeneic Hematopoietic Stem Cell Transplantation
LSCs: Leukemic Stem Cells
BM: Bone Marrow
BMME: Bone Marrow microenvironment
HMAs: Hypomethylating Agents
CMPs: Common Myeloid Progenitors
GMP: Granulocyte-Monocyte Progenitor
CH: Clonal Hematopoiesis
BMSCs: Bone marrow stromal cells
MSCs: Mesenchymal Stem Cells
IL: Interleukin
MDSCs: Myeloid-Derived Suppressor Cells
PD-1: Programmed Death-1
DC: Dendritic Cells
HIF-1: Hypoxia-Inducible Transcriptional Factors-1
mTOR: Mechanistic Target of Rapamycin
CHIP: Clonal Hematopoiesis of Indeterminate Potential
TIM3: T-cell Immunoglobulin and Mucin Domain 3
Gal-9: Galectin-9
CEACAM1: Carcinoembryonic Antigen-Related Cell Adhesion Molecule 1
ROS: Reactive Oxygen Species
OS: Oxidative Stress
HMAF: Hypomethylating Agent Failure
